# Improving the visual communication of environmental model projections

**DOI:** 10.1038/s41598-021-98290-4

**Published:** 2021-09-27

**Authors:** Hayley J. Bannister, Paul G. Blackwell, Kieran Hyder, Thomas J. Webb

**Affiliations:** 1 Department of Animal and Plant Sciences, University of Sheffield, Sheffield, S10 2TN UK; 2 School of Mathematics and Statistics, University of Sheffield, Sheffield, UK; 3 Centre for Environment, Fisheries and Aquaculture Science, Lowestoft, UK; 4 School of Environmental Sciences, University of East Anglia, Norwich, UK

**Keywords:** Environmental impact, Ecological modelling

## Abstract

Environmental and ecosystem models can help to guide management of changing natural systems by projecting alternative future states under a common set of scenarios. Combining contrasting models into multi-model ensembles (MMEs) can improve the skill and reliability of projections, but associated uncertainty complicates communication of outputs, affecting both the effectiveness of management decisions and, sometimes, public trust in scientific evidence itself. Effective data visualisation can play a key role in accurately communicating such complex outcomes, but we lack an evidence base to enable us to design them to be visually appealing whilst also effectively communicating accurate information. To address this, we conducted a survey to identify the most effective methods for visually communicating the outputs of an ensemble of global climate models. We measured the accuracy, confidence, and ease with which the survey participants were able to interpret 10 visualisations depicting the same set of model outputs in different ways, as well as their preferences. Dot and box plots outperformed all other visualisations, heat maps and radar plots were comparatively ineffective, while our infographic scored highly for visual appeal but lacked information necessary for accurate interpretation. We provide a set of guidelines for visually communicating the outputs of MMEs across a wide range of research areas, aimed at maximising the impact of the visualisations, whilst minimizing the potential for misinterpretations, increasing the societal impact of the models and ensuring they are well-placed to support management in the future.

## Introduction

Understanding the likelihood of alternative future states is a key challenge for managing natural systems in the face of global change. A wide range of environmental models have been used to help develop management solutions, but the differing structures and assumptions of individual models can lead to wildly different predictions^1,2^. An increasingly popular way to deal with the shortcomings of individual models is to combine the outputs of multiple structurally different models that have been run under a common set of scenarios for the future into a Multi-Model Ensemble (MME). MMEs have been successfully used to increase the skill and reliability of model predictions in a wide range of research areas, including climate science (e.g.^3,4^) and terrestrial and marine ecosystem modelling (e.g.^5,6^). However, these increases in skill and reliability can come at the cost of seemingly greater uncertainties in the outputs as, for example, different models within the ensemble may give contrasting predictions for the future^1^. In the past, a lack of effective communication of such variable model outputs, both to decision makers and the general public, has been blamed for ineffective management decisions^7^. This in turn has contributed to public distrust of scientific evidence, particularly in regards to climate science^8,9^. Improving the communication of uncertainties to non-specialist audiences is therefore vital to ensuring that environmental models continue to make a significant contribution to the decision-making process^10,11^.

Well-designed data visualisations tend to resonate particularly well with non-specialist audiences, especially on social media platforms where they can be easily shared between a large network of individuals. A recent example of the successful communication of climate science involves the very popular ‘show your stripes’ visualisation (showyourstripes.info), which was widely shared on television and Twitter. Although data visualisation represents a powerful method for improving communication, little research has been conducted to identify the most effective methods for visually communicating the uncertainties associated with complex environmental models^12–14^. As a result, many of the visualisations used in environmental modelling ignore the presence of uncertainties or are used to depict only one source of uncertainty at a time^13,15,16^. This is particularly problematic when attempting to communicate the outputs of MMEs, which typically require a visual representation of changes in both model and scenario uncertainties over time. Whilst animated and interactive visualisations could be used to communicate multiple uncertainties at the same time, these methods are not appropriate for media requiring static images, and interactive visualisations may require a greater level of skill or expertise to use than static or animated visualisations^14^. By focusing on how best to communicate the outputs of MMEs using static visualisations, we may be able to improve engagement and trust across a broader cross-section of society than would be possible using animated or interactive visualisations.

Examples of static visualisations that are often used to communicate uncertainty in environmental modelling include line, dot, and box plots. These visualisation methods typically depict a summary of the data, such as an average, and an estimate of the uncertainty through the use of uncertainty bands (or envelopes) or error bars. Although dot and box plots have previously been shown to be effective at communicating uncertain snowfall forecasts^17^, relatively little is known about the ability of the general public to interpret these visualisations. It is also possible that more modern visualisation methods, such as infographics and cascade plots (e.g.^18,19^), may outperform dot and box plots when used to communicate the outputs of MMEs to decision makers and/or the general public. Traditional methods of visualisation that are less frequently used in environmental modelling, such as radar (or spider) plots and heat maps, may also perform well when adapted to depict the outputs of MMEs. However, there is a lack of empirical research comparing the performance of these visualisation methods when used to communicate the outputs of MMEs to different groups of people^20^, making it difficult for researchers to maximise the impact of these model ensembles.

Various methods may be used to assess the performance of different methods of visualising uncertainty (see^21^ for a review). Typically, the effectiveness of a visualisation is determined by measuring the accuracy and/or self-assessed confidence with which a set of individuals are able to interpret the visualisation^20,21^. User preferences and subjective measures of ease of use are also often used to compare the performance of different visualisation methods^21^. However, we are not aware of any research that has combined all these measures of performance to determine the most effective methods for visually communicating the outputs of MMEs. We aim to fill this research gap by conducting an in-depth online survey that measures the accuracy, confidence, and ease with which the participants are able to interpret 10 different visualisations, all of which depict the same set of data from a well-established climate MME (https://pcmdi.llnl.gov/mips/cmip5/), as well as their subjective preferences for each of the visualisations. As the effectiveness of each visualisation method may depend on factors such as the numeracy and scientific literacy of the audience^14^, we also take into account the education level, background, and expertise of the participants when determining the performance of each visualisation. The results are used to generate guidelines for visually communicating the outputs of MMEs across a wide range of different research areas. These guidelines can be used to maximise the impact of the visualisations, whilst minimising the potential for misinterpretations. This in turn will help to increase the societal impact of the models and ensure they are well-placed to support management in the future.

## Methods

To better understand the effectiveness of different methods of visualising MMEs, we developed a series of visualisations of projections of global surface air temperature (referred to simply as ‘temperature’ from here on) from the climate MME produced during phase five of the Coupled Model Intercomparison Project (CMIP5) (https://pcmdi.llnl.gov/mips/cmip5/).

### The models

We extracted annual mean temperature projections from the IPCC AR5 database (﻿dkrz.de) for 15 different models. Each model simulated historical temperatures between 1850 and 2005 and three greenhouse gas emissions scenarios, known as Representative Concentration Pathways (RCPs), between 2006 and 2100. The three RCPs, referred to as RCP 2.6 [low emissions], RCP 4.5 [intermediate emissions], and RCP 8.5 [high emissions], span the range of currently available estimates for the predicted level of radiative forcing that is expected to occur by the end of the century^22^. Although some of the models were run under each scenario multiple times, we chose to use only one set of outputs per model to ensure all models were treated equally. The projected change in temperature expected in each year was quantified by comparing the model outputs under each RCP scenario with the mean temperature during a pre-industrialisation reference period (1850–1900).

### The visualisations

**Figure 1:**
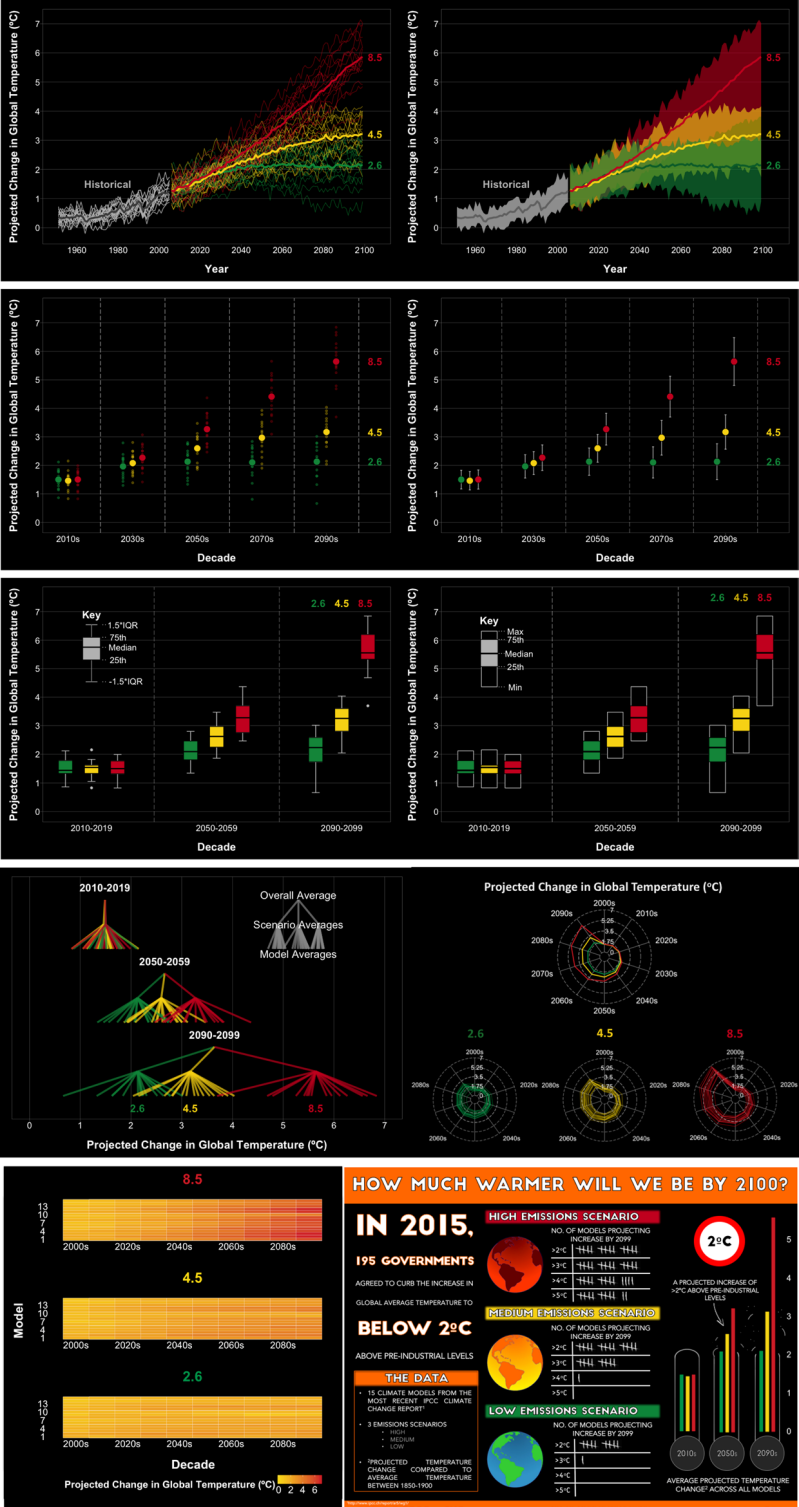
The ten visualisations included in the survey. From left to right and top to bottom: the line1, line2, dot1, dot2, box1, box2, cascade, radar, heat map and infographic plots. Each visualisation was accompanied by a legend with a minimal description of the content of the visualisation alongside definitions of any statistical terminology used.

The model outputs were used to develop ten visualisations that depict the same data in different ways: two line plots (line1 and line2), two dot plots (dot1 and dot2), two box plots (box1 and box2), a radar plot, a cascade plot, a heat map, and an infographic (Fig. 1). These visualisation methods were chosen to represent plots that are frequently used in the scientific literature and the media, as well as some that are more unusual and may be less familiar to a wider audience. All visualisations depicted the data in at least three decades: the 2010s, the 2050s, and 2090s. The culturally ingrained traffic light colour system (i.e. red, amber, green) was used in all ten visualisations to maintain consistency. However, the selected colour scheme may make it more difficult for those who experience deuteranopia or protanopia to distinguish between the different colours. For this reason, we added the option for the participants to request visualisations with a more suitable colour palette if required, although none of the participants selected this option.

### Survey demographics

The survey received full ethical approval from the University of Sheffield’s Department of Animal and Plant Sciences, in accordance with the University of Sheffield’s Research Ethics Approval Procedure. The survey was performed in accordance with the relevant guidelines and regulations and informed consent was obtained from all participants.

380 participants took part in the survey; the majority were aged between 18 and 34 (n = 258), 96 were aged between 35 and 54, and relatively few were over the age of 55 (n = 26). There were slightly more female participants than males (n = 202 and n = 174 respectively) and most participants were based in the United Kingdom (n = 306), although individuals from a total of 21 countries participated in the survey. 318 participants held a university-level qualification, whilst 60 participants held GCSE, A-Level, or vocational qualifications. 18 participants considered themselves to be a decision maker or environmental manager and 190 considered themselves to be a scientist. 131 participants had previously worked with environmental models and/or their outputs to some extent, although not all of these considered themselves to have a good level of expertise: overall 285 of the 380 participants considered themselves to have little to no expertise in working with environmental models and/or their outputs. 49 participants had five or more years of experience, whilst 44 considered themselves to be an expert.

### The survey

The survey was developed using the Qualtrics online survey software (qualtrics.com). To test the effectiveness of individual visualisations, participants were shown a randomly selected visualisation (A) and asked if they had encountered a similar visualisation prior to completing the survey. Next the participants were asked to estimate the average, minimum, and maximum global temperature change projected to occur in a randomly selected decade and scenario (if possible using the visualisation provided). We used the term ‘average’ instead of ‘mean’ to ensure the survey remained accessible to a wider audience. Each participant was then required to comment on the confidence and ease with which they were able to interpret the visualisation, on a Likert scale (disagree, somewhat disagree, neutral, somewhat agree, agree).

To test for preferences between different visualisations, the participants were then shown a second randomly selected visualisation (B) alongside the first and asked to choose which of the two visualisations they preferred across five different categories: the ability to view changes in temperature over time, the ability to view changes in uncertainty over time, the ability to retrieve specific values (such as the mean), visual appeal, and overall ease of understanding. The participants were given the option of preferring visualisation A, preferring visualisation B, or having no preference for either A or B. In cases where the participant showed no preference, they were given the option of selecting ‘both the same’ or neither.

The survey was designed so that the participants could choose to complete the above tasks up to five times using different sets of randomly selected visualisations. A full example of the questions asked in the survey is provided in Supplementary Methods 1.

### Statistical analysis

To better understand the accuracy of participants' interpretation of visualisations, we used Generalised Linear Mixed Models (GLMMs)^23^ to model the absolute differences between participants’ estimates of the mean, minimum, and maximum projected temperature change and the ‘true’ values given by the climate models. The results are presented as predicted ratios between the inaccuracies associated with the ‘typical’ response and the inaccuracies associated with all other levels of the predictor variables (referred to as Absolute Difference (AD) ratios from here on), thereby allowing an assessment of the variability in the differences in accuracy across all levels of the predictor variables.

The typical participant was asked to estimate the mean, minimum, and maximum temperature change projected to occur in the 2050s in scenario 4.5 using the box1 plot. As there were no ‘typical’ visualisations in the survey, the box1 plot was selected as it tended to fall in the middle of the visualisations when ranked based on the mean absolute difference between the participants’ estimates of the mean, minimum, and maximum projected temperature change and the true value given by the climate models. It is important to note that because the results of the analysis are presented relative to the ‘typical’ response, they apply only when all other predictor variables are fixed at the reference levels.

An AD ratio greater than one suggests the participants in the group in question tended to be less accurate than the reference group and vice versa. As there are no widely accepted methods for incorporating the uncertainty in the random effects at present^24^, the standard errors (and hence 95% confidence intervals) of the predictions should be treated as lower bounds of the uncertainty.

To analyse the participants’ views on confidence and ease, we used Mixed Partial Proportional Odds Models (MPPOMs)^25,26^. These are regression models with ordinal responses, with fixed and random effects (as described below), and with a mixture of proportional-odds and nominal covariates; any explanatory variables that did not meet the assumption of proportional odds^26,27^ were treated as nominal effects (see Supplementary Methods 2). As above, the model outputs are presented relative to the ‘typical’ response, which was based on the ‘average’ participant. In this case, the average participant was a postgraduate scientist with no experience in working with environmental models and/or their outputs.

Both the GLMMs and the MPPOMs included visualisation type, decade, scenario, background, level of education, and length of expertise in working with environmental models and/or their outputs as fixed effects. The MPPOMs additionally included previous encounters as a fixed effect. Participant ID was included as a random effect in both model types to take into account the fact that the participants were able to answer the same set of questions with different visualisations up to five times. As there are no widely accepted measures of model fit for quasi-methods or MPPOMs, we fit the full models (minus the interactions) instead of searching for the best-fitting models.

To analyse participants’ preferences for different visualisations, we used Bradley-Terry `contest' models^28^, in which preference is seen as a contest between the two visualisations involved, but may be tied if the participant expresses no preference. Participants that considered themselves to be decision makers and/or environmental managers were removed from the main part of the analysis due to small sample sizes causing issues with model fit. The best-fitting Bradley-Terry models were selected using Akaike’s Information Criterion (AIC)^29^. A predicted preference score was extracted for each visualisation, with a greater score indicating that the visualisation was preferred more often than one with a lower score. 95% ‘comparison’ intervals (which are based on ‘quasi’ standard errors) were used to allow us to compare across all visualisations rather than making comparisons with only the reference level^28^.

The parameter estimates of the above statistical models were used to rank each of the visualisations based on accuracy, confidence/ease, and preference. The final rankings in each category were aggregated to produce an overall ranking for each visualisation using the Cross-Entropy Monte Carlo algorithm^30^. This algorithm searches for the final ranking that minimises the ‘distance’ between itself and the original rankings, where distance is measured using Kendall’s tau^31^.

All statistical analyses were performed in R using the MASS^32^, ordinal^25^, BradleyTerry2^28^, qvalc^33^ and RankAggreg packages^34^.

## Results

### Previous encounters

The participants were most familiar with the box1 plot, with over 70% of the participants having previously encountered a similar visualisation (Supplementary Figure 1). Over 50% of the survey participants had also encountered visualisations similar to the dot2, line1, and line2 plots (Supplementary Figure 1). The cascade plot was by far the least familiar of all the visualisations in the survey, with over 90% of the participants having not seen a similar visualisation in the past (Supplementary Figure 1). Over 50% of the survey participants had also not previously encountered visualisations that were similar to the heat map or infographic (Supplementary Figure 1).

### Accuracy

Visualisation type had a significant effect (*p* < 0.05) on all three measures of accuracy. In all cases, participants were more accurate when using the dot1 plot compared with the reference box1 plot (and hence all other visualisations that performed worse than the box1 plot), while the opposite was true for the heat map. More specifically, the AD ratio of the dot1 plot typically fell below 0.75, whilst the AD ratio of the heat map was consistently closer to 2 (Supplementary Figure 2). Furthermore, the infographic was associated with greater inaccuracies than the reference box1 plot (and hence all other visualisations that outperformed than the box1 plot) when the participants were asked to estimate the minimum and maximum, with AD ratios (95% CI) of 1.59 (1.24, 2.04) and 2.07 (1.58, 2.71) respectively (Supplementary Figure 2). Interestingly, the dot2 plot displayed a similar level of accuracy to the dot1 plot when the participants were asked to estimate the mean but performed comparatively poorly when they were asked to estimate the maximum, with AD ratios (95% CI) of 0.68 (0.50, 0.91) and 1.20 (0.92, 1.56) respectively (Supplementary Figure 2).

The background of the participant also had a significant effect (*p* < 0.05) on the accuracy with which they were able to estimate the mean, minimum, and maximum. For example, decision makers/environmental managers and the general public were less accurate than the reference scientists when they were asked to estimate the mean, with AD ratios (95% CI) of 2.09 (1.35, 3.24) for decision makers/environmental managers and 1.56 (1.22, 2.00) for the general public (Supplementary Figure 2). Conversely, the scenario and decade given to each participant, as well as their level of education and expertise in working with environmental models and/or their outputs, had comparatively little effect on the accuracy with which they were able to interpret the visualisations.

### Confidence and ease

**Figure 2:**
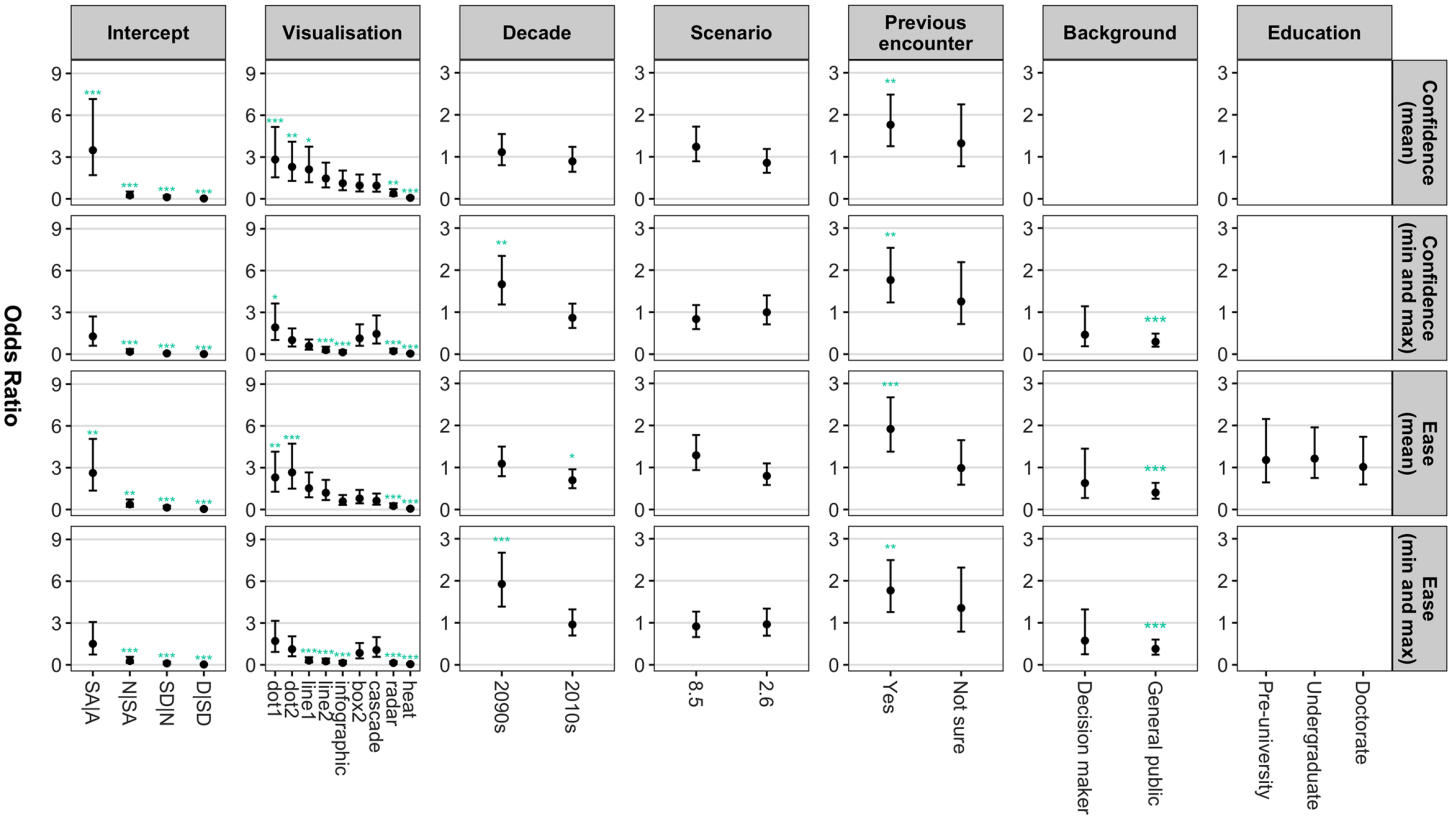
The regression coefficients (or 'odds ratios') (95% confidence interval) of the MPPOMs that were used to analyse the confidence and ease with which the participants were able to estimate the mean and minimum/maximum projected temperature change. The levels of the predictor variables that were included in the ‘typical’ response are included in the coefficients associated with the intercept (bottom). SA|A refers to the threshold between somewhat agree and agree, N|SA refers to the threshold between neutral and somewhat agree and so on. Please note the differences in the y-axis limits. A blank space indicates the predictor variable was included in the MPPOM as a nominal effect. *p < 0.05, **p < 0.01, ***p < 0.001.

**Figure 3:**
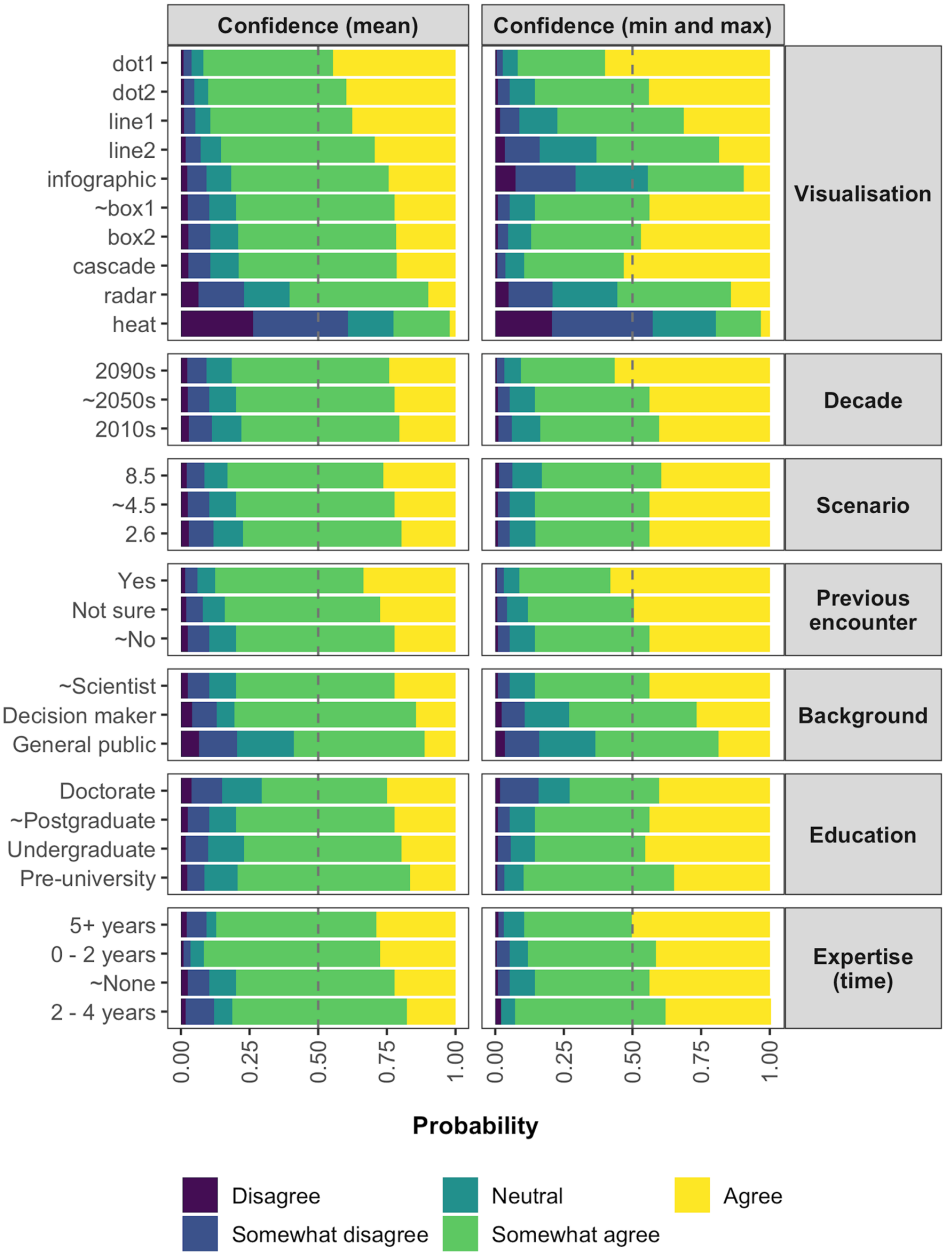
The predictions of the MPPOMs that were used to analyse the confidence with which the participants were able to estimate the mean (left) or minimum/maximum (right) projected temperature change. The predictions are given as probabilities for each level of the Likert scale, ranging from ‘disagree’ in purple to ‘agree’ in yellow. The reference levels associated with the ‘typical’ response are marked with a tilde.

Visualisation type had a significant effect (*p* < 0.05) on all measures of the confidence and ease with which respondents were able to interpret the visualisation. For example, the participants were up to 2.82 (95% CI 1.54, 5.16) times more likely to select a higher rating for confidence and ease when they were asked to estimate the mean using the dot plots compared with the reference box1 plot (Fig. 2). On the other hand, the participants were less likely to select a higher rating for confidence and ease when they were asked to estimate the mean or the minimum/maximum using the radar plot and heat map, both of which had odds ratios (95% CI) of less than 0.39 (0.21, 0.70) (Fig. 2). The same was also true when the participants were asked to estimate the minimum and maximum using the line1, line2 and/or infographic plots (Fig. 2). To further illustrate these results, the probability of a positive confidence rating (i.e. agree or somewhat agree) reached highs of 0.92 when the participants were asked to estimate the mean using the dot plots, but this dropped to 0.61 and 0.23 when they were asked to interpret the radar plot and heat map respectively (Fig. 3). For the infographic and line2 plot, the probability of a positive confidence rating decreased from 0.82 and 0.85 for the mean to 0.44 and 0.63 for the minimum/maximum respectively (Fig. 3).

The familiarity of the visualisation also had a significant effect (*p* < 0.01) on both the confidence and ease with which the participants were able to interpret the visualisations (Fig. 2). More specifically, the participants were up to 1.92 (95% CI 1.38, 2.67) times more likely to select a higher rating for confidence and ease when they were asked to interpret a visualisation they were familiar with compared with a visualisation type they had never previously encountered (Fig. 2). Likewise, members of the general public were typically less likely to select one of the higher Likert scale ratings compared with the reference scientists, with odds ratios (95% CI) of between 0.30 (0.18, 0.49) and 0.40 (0.26, 0.63) (Fig. 2). The most dramatic difference between the reference scientists and the general public occurred when the participants were asked to comment on the confidence with which they were able to estimate the minimum and maximum; the predicted probability of a scientist giving a positive rating was 0.85, whilst the probability of a member of the general public doing the same was just 0.63 (Fig. 3). Conversely, the probability of a positive rating for confidence was relatively consistent across all decades, scenarios, levels of education, and levels of expertise.

### Preference

The two line plots performed particularly well when used to view changes in the mean over time; these visualisations had preference scores that were significantly greater (*p* < 0.001) than the box1 plot (and hence the radar, heat map, infographic, and cascade plots), with scores (95% comparison intervals) of up to 1.24 (0.82, 1.66) (Supplementary Figure 3). Conversely, the participants displayed the greatest preference for the two box plots when used to retrieve specific values. In this example, the preference scores of the two box plots were significantly greater (*p* < 0.01) than the preference scores of all other visualisation types excluding the two dot plots (Supplementary Figure 3). Both the dot and box plots also performed well when used to view changes in uncertainty over time, with preference scores (95% comparison interval) of up to 0.1 (− 0.29, 0.48) compared with a preference score of − 2.41 (− 2.91, − 1.9) for the worst performing visualisation (the heat map) (Supplementary Figure 3).

On the other hand, the preference scores of the dot plots were significantly greater (*p* < 0.05) than the reference box1 plot (and hence all other visualisations with lower preference scores) for several preference categories, including visual appeal and overall ease of understanding (Supplementary Figure 3). For example, the preference score (95% comparison interval) of the dot2 plot was 1.27 (0.37, 2.17) for overall ease of understanding, a value that was significantly greater (*p* < 0.05) than all the other visualisations excluding the box2 plot (Supplementary Figure 3).

Generally, the heat map, radar, infographic, and cascade plots were the least preferred visualisations across all preference categories, with the heat map performing particularly poorly. However, there were some notable exceptions. For example, the cascade plot performed well when used to view changes in uncertainty over time, with a preference score that was in line with those of the line, dot, and box plots. Similarly, the infographic performed comparatively well for visual appeal, although there was a great deal of overlap between the preference scores of all the visualisations for this preference category.

### Rankings

**Figure 4:**
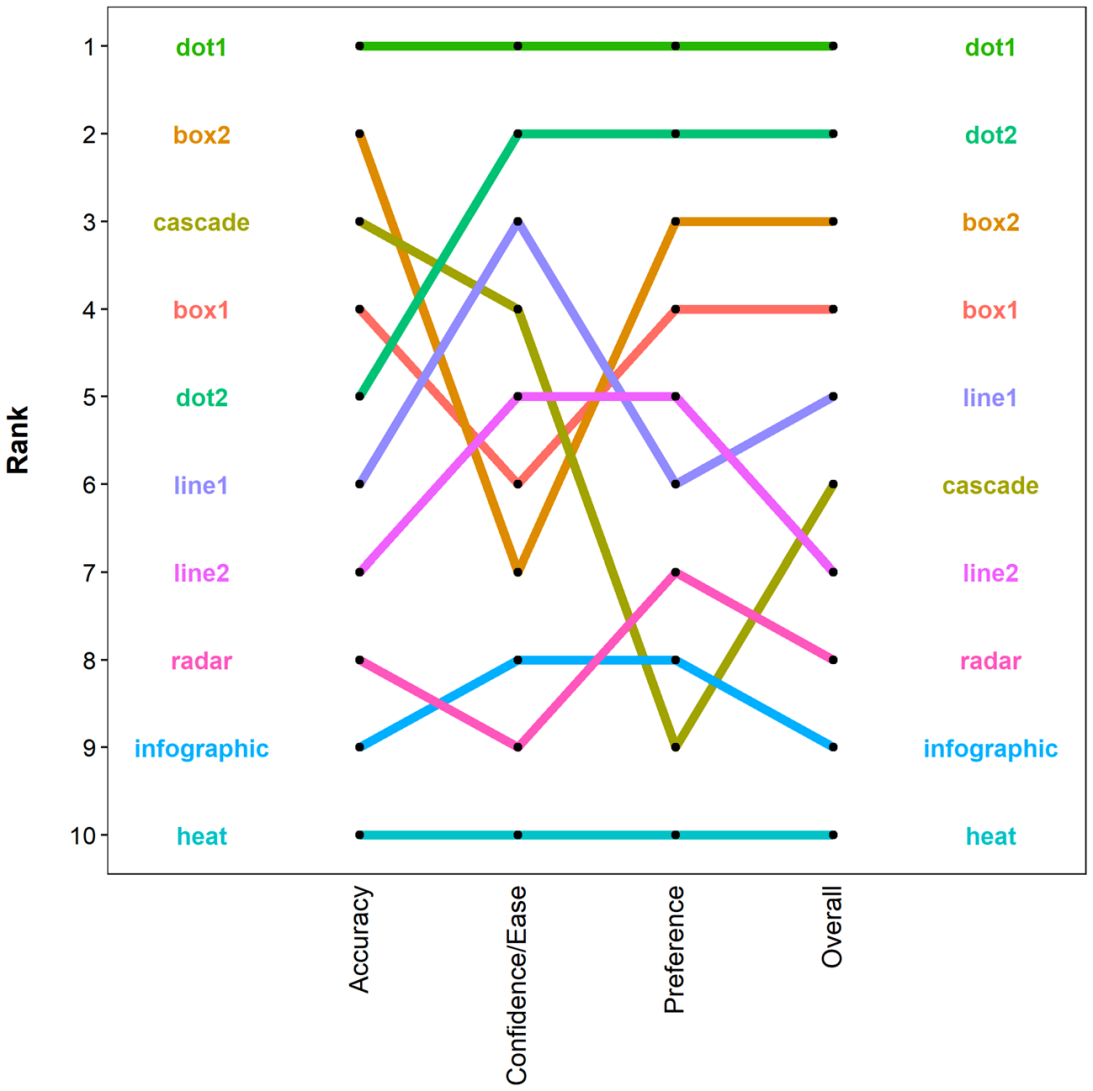
The rankings of the visualisations based on participant accuracy, confidence/ease, and preferences. An 'overall' ranking is given based on the aggregation of the rankings for each of these categories.

The dot1 plot consistently ranked in first position across all measures of performance, while the heat map consistently ranked last (Fig. 4). The dot2 plot performed relatively poorly for accuracy but was second best for confidence/ease and preferences, thus resulting in an overall ranking of second (Fig. 4). Both of the box plots were in the top four for accuracy and preferences but dropped down to sixth and seventh position for confidence/ease (Fig. 4). However, this drop in performance had little effect on the overall rankings of the box plots, which placed in third and fourth (Fig. 4). The cascade plot was ranked in third and fourth for accuracy and confidence/ease respectively, but in ninth for preferences, resulting in an overall ranking of sixth. The line plots displayed an intermediate level of performance across all categories, remaining between third and seventh throughout (Fig. 4). Finally, the radar plot and infographic were ranked in the bottom four across all categories (Fig. 4).

## Discussion

### Visualisation type

In our online survey, the type of visualisation that was shown to the participants had a significant effect on the accuracy, confidence, and ease with which they were able to interpret the outputs of the MME. The dot plots outperformed all other visualisation types across almost all measures of performance. However, the participants struggled to estimate the minimum and maximum using the dot2 plot, likely due to the uncertainty being represented using error bars that were determined using the standard deviation of the projections. Nevertheless, the dot2 plot ranked highly when used to view changes in uncertainty over time and to retrieve specific values, and fewer than 10% of the participants correctly identified that they were unable to accurately estimate the minimum and maximum. Those participants that did try to give an estimate may have misinterpreted what the error bars represented. Such misinterpretations have been widely recognized (e.g.^35–37^), with some researchers suggesting that error bars may in fact be harmful^38^. This finding is supported by our results, which showed that the participants’ ratings of confidence and ease remained relatively high despite the drop in accuracy. The dot2 plot may therefore be unintentionally misleading, a potentially dangerous trait given that it is likely to result in an underestimation of the uncertainty in the model outputs. A similar issue might also be expected to affect the box1 plot, which included error bars that represented the minimum and maximum that was within 1.5 × the interquartile range. However, this was not the case, likely due to the relatively small difference in the upper and lower limits of the error bars and the ‘true’ minimum and maximum.

Despite the use of error bars, the dot and box plots were ranked in the top four overall. Ibrekk and Morgan^17^ also found that survey participants were most accurately able to interpret uncertain snowfall forecasts when using a dot or box plot, although none of the visualisations that were tested (a bar chart, a pie chart, and several different representations of probability and cumulative distribution functions) depicted more than one type of uncertainty. Our results therefore extend the work of^17^ by highlighting the effectiveness of the dot and box plots across a much wider audience (their survey consisted of 45 individuals from a single environmental education facility), across a greater number of measures of performance, and when communicating more than one type of uncertainty.

The success of these visualisations is supported by^20^, who found that visualisations containing limited summary information were deemed to be more useful than those containing more information (e.g. the line plots). In fact, the two line plots consistently displayed an intermediate level of performance, which is perhaps surprising given these visualisations are generally believed to be easy to interpret and have previously proven to be successful at conveying trends in environmental variables over time^39,40^. However, the participants struggled to quantify the uncertainty when using the line plots, likely as a result of over-plotting, which can make it more difficult or even impossible to identify specific values^41^. This may also explain why the line plots performed relatively poorly across many of the preference categories, particularly the ability to view changes in uncertainty over time and the ability to retrieve specific values. This theory is supported by a similar survey conducted by^42^, which suggested that over-plotting may have impacted the participants’ ability to assess future changes in rainfall. Line plots may therefore perform better in situations where there are fewer model runs to display or when the scenarios diverge more dramatically.

Aspect ratios also have an important impact on the interpretability of line plots as greater aspect ratios may emphasise larger-scale trends, whilst hiding small-scale features^43^. Although it is possible the aspect ratio of the line plots may have impacted interpretability during the survey, we consistently used the same aspect ratio across all of the line, dot and box plots and so it is unlikely to be the sole cause of the comparatively poor performance of the line plots. Nevertheless, we recommend investigating whether optimisation techniques such as ‘multi-scale banking to 45°’^43^ help to improve the interpretability of MME time-series plots in future. A different colour scheme designed to maximise contrast between colours, rather than to match the culturally ingrained ‘traffic light’ system, coupled with bolder lines may further help to improve the performance of the line plots.

The cascade plot showed perhaps the greatest variation in rankings across the different measures of performance, placing in third and fourth for accuracy and confidence/ease respectively, but in ninth for preference. It is likely that the cascade plot performed poorly across many of the preference categories as preferences have often been shown to be strongly related to familiarity (e.g.^44–46^). As the cascade plot was the least familiar visualisation, it is more likely to be one of the least preferred visualisations. Increased exposure to this type of visualisation may therefore help to improve the performance of the cascade plot, particularly in terms of overall ease of understanding and visual appeal.

The radar plot performed poorly across all measures of performance, with rankings consistently at or below seventh. Such poor performance is expected given that radar plots are typically used to display multivariate data^47,48^, rather than to communicate changes in a single environmental variable through time. Similar to the cascade plot, the radar plot was also one of the most unfamiliar visualisations and would therefore be more likely to be one of the least preferred visualisations. Furthermore, the cognitive load (or mental effort) required to interpret the radar plot may be far greater than for many of the other visualisation types due to the axes pointing in different directions^49^. As increased cognitive loads have previously been shown to have a negative impact on the accuracy with which individuals may interpret a visualisation^50^, this issue may help to explain the negative response of the participants to the radar plot.

The infographic was consistently found in the bottom three. However, it is likely the infographic performed poorly as we purposefully chose not to provide enough information in this visualisation to enable accurate interpretation. Despite this, only 40% of the participants recognized that they were unable to estimate the minimum and/or maximum. This suggests that either the participants misinterpreted the visualisation or there was some ambiguity in the phrasing of the question that resulted in a different interpretation than what was intended. Interestingly, the infographic still ranked relatively highly for visual appeal, highlighting the power of this type of visualisation to grab the attention of an audience despite not necessarily communicating the message clearly. The potential for misinterpretations has important implications for the use of infographics in the communication of MMEs in the future; each infographic must be carefully designed to ensure the visualisation is engaging but that the intended message is delivered with clarity and integrity. Achieving a balance between these factors and visual appeal may be best achieved through interdisciplinary collaborations between natural scientists, social scientists, and graphic designers or communication experts^51^. The end users should also be included in such collaborations to ensure the impact of the visualisation can be maximized, whilst misinterpretations can be minimised^51^.

Finally, the heat map was the weakest visualisation across all measures of performance. Such poor performance may be driven by the difficulties associated with extracting specific values from a continuous scale bar^52^. This issue may also be exacerbated by the use of a sequential colour scale with relatively little variation in colour between the minimum and maximum. A diverging colour scheme may have improved the performance of the heat map by dividing the scale bar into three easily identifiable regions (low, medium, high), thus providing more visual cues with which to interpret the visualisation^53^.

### Familiarity and background

The familiarity of the visualisations had a significant effect on the confidence and ease with which the participants were able to interpret the visualisations. This is unsurprising given that the participants would likely be better equipped to interpret a visualisation they had previously encountered compared with one they had never seen before. As previously mentioned, numerous studies have also linked familiarity with visualisation preferences, particularly in terms of visual appeal (e.g.^42,54,55^). Despite this, the final rankings of the visualisations did not follow the ordering of the visualisations that were most familiar to the survey participants. For example, the dot1 plot performed well across all measures, but was less familiar than the dot2, box, and line plots. However, the similarities between the dot1 and dot2 plots may have made it easier for the participants to interpret the dot1 plot compared with a visualisation that showed no similarities to any of the more familiar visualisations. The cascade plot was also the least familiar visualisation, but it performed well for accuracy, confidence, and ease; this is important as it proves that an unfamiliar visualisation can outperform traditional visualisation types if used correctly. Nevertheless, the cascade plot performed comparatively poorly for preferences, particularly for ease of understanding and visual appeal, thus supporting the conclusion that familiarity is at least somewhat related to preferences.

Background also had a significant effect on the ability of the participants to complete the survey. More specifically, decision makers, environmental managers, and the general public tended to be less accurate, less confident, and find it more difficult to interpret the visualisations than scientists. This is to be expected given that scientists likely spend far more time creating and interpreting visualisations similar to those in the survey. This result emphasises the differing needs of non-specialist audiences, something that is often not explored in studies focusing on uncertainty visualisation (e.g.^17,20^). However, it is important to note that the uncertainty surrounding the estimates of the decision makers and environmental managers were often relatively large due to the small sample size of this group. A much larger sample size would be required to make robust conclusions about the ability of this group of individuals to interpret these visualisations, as well as their preferences for different visualisations.

### Recommendations

Based on the findings of this research, as well as the extended feedback provided by the participants, we recommend the following when visualising the outputs of MMEs:

1. Think carefully about the intended audience of the visualisation. If communicating to non-specialist audiences, consider adding more descriptive labels to the visualisation. If possible, provide a detailed description of what the visualisation represents and include specific values that may be important. The term ‘average’ may be used for accessibility, but this should be followed by a statement that indicates whether this represents the mean or the median to avoid confusion.
2. Consider using both familiar (e.g. dot, line, and box) and unfamiliar (e.g. cascade) visualisation types. Familiar visualisations may maximise uptake, but new methods of visualisation may outperform more familiar techniques in some instances. Nevertheless, new methods should be widely tested before implementation.
3. Consider using dot or box plots when developing visualisations that require the users to extract specific values from the visualisation.
4. If using infographics, ensure they are designed in a way that balances visual appeal with clarity and integrity.
5. Use line plots only when over-plotting is unlikely to be an issue.
6. Do not use radar plots when attempting to communicate changes in a single environmental variable through time.
7. Do not use heat maps that have a sequential colour scale with relatively little variation in colour between the minimum and maximum values if you want users to be able to extract specific values from the visualisation.
8. Exercise caution when using error bars. Label the error bars carefully and provide a detailed description of what the error bars represent.
9. Choose colour schemes wisely. Avoid using colour schemes that might be difficult to interpret for those who experience any form of colour vision deficiency. Avoid using a black background if using other colours that will be difficult to distinguish against a dark background.

Despite these recommendations, it is important to note that the visualisations that did not perform well in the survey may be useful for other purposes, and the specific visualisations that we selected may not be optimal in all contexts. However, our results do reveal general conclusions about the attributes that make a visualisation effective—information that will enable the development of suitable visualisations for specific audiences and purposes. Doing so will enable us to target audiences with visualisations that both capture their interest and prevent misinterpretations of the data, as well as help to increase the societal impact of the models and ensure they are well-placed to support management decisions in the future.

## Supplementary Information

Supplementary Information.
