# Supplementary material for: Improving the visual communication of environmental model projections
Source: Sci Rep. 2021 Sep 27;11:19157. doi: 10.1038/s41598-021-98290-4 (PMC8476535; doi:10.1038/s41598-021-98290-4)
Supplement: Supplementary file 1 — Supplementary Information. [file 41598_2021_98290_MOESM1_ESM.pdf]

**Improving the visual communication of environmental model projections**

**Supplementary information**

Hayley J. Bannister[1,2,3*], Paul G. Blackwell[2], Kieran Hyder[3,4] and Thomas J. Webb[1]

[1]Department of Animal and Plant Sciences, University of Sheffield, Sheffield, UK

[2]School of Mathematics and Statistics, University of Sheffield, Sheffield, UK

[3]Centre for Environment, Fisheries and Aquaculture Science, Lowestoft, UK

[4]School of Environmental Sciences, University of East Anglia, Norwich, UK

*Hayley J. Bannister, Department of Animal and Plant Sciences, University of Sheffield, Sheffield, S10 2TN, UK.

**Telephone**: (+44)7568 199959

**Email:** hayleyjbannister@gmail.com

**Supplementary Methods 1: Example survey**

**Prior to completing this survey, had you encountered a similar visualisation to the one shown above?**
Yes
No
Not sure

**Using the above visualisation:**

**Estimate the average global temperature change projected to occur by the end of the 2090s under Scenario 8.5 (high emissions).**
[Sliding bar from 0 to 7°C with an accuracy of 1 decimal place]

**Please indicate the extent to which you agree or disagree with the following statements in regards to the question above:**

**I am confident in the answer I provided.**
Disagree
Somewhat disagree
Neutral
Somewhat agree
Agree

**I found it easy to identify the answer.**
Disagree
Somewhat disagree
Neutral
Somewhat agree
Agree

**What other information would you need to improve your ability to estimate the average global temperature change projected to occur by a given point in time?**
[Empty text box for response]

**What is the level of uncertainty surrounding the average global temperature change projected to occur by the end of the 2090s under Scenario 8.5 (high emissions)?**
Very low
Low
Moderate
High
Very high

**If possible, please provide an estimate of the range of global temperature changes projected to occur by the end of the 2010s under Scenario 2.6 (low emissions).**
[Two sliding bars from 0 to 7°C with an accuracy of 1 decimal place - one for the lowest possible change and one for the highest possible change. A 'Not Applicable' option was provided for both.]

**Please indicate the extent to which you agree or disagree with the following statements in regards to the question above:**

**I am confident in the answer I provided.**
Disagree
Somewhat disagree
Neutral
Somewhat agree
Agree

**I found it easy to identify the answer.**
Disagree
Somewhat disagree
Neutral
Somewhat agree
Agree

**What other information would you need to improve your ability to estimate the range of global temperature changes projected to occur by a given point in time?**
[Empty text box for response]

**The visualisation shown below (B) represents another option that may be used to present the same data as the previous visualisation (A).**

**Please indicate your preference for one (or both) of the visualisations in the following aspects:**

**Ability to view changes in temperature over time.**
A (the first visualisation)
B (the second visualisation)
Both the same
Neither

**Ability to view changes in uncertainty over time.**
A (the first visualisation)
B (the second visualisation)
Both the same
Neither

**Ability to retrieve specific values (e.g. mean, minimum, maximum)**
A (the first visualisation)
B (the second visualisation)
Both the same
Neither

**Visual appeal**
A (the first visualisation)
B (the second visualisation)
Both the same
Neither

**Overall ease of understanding**
A (the first visualisation)
B (the second visualisation)
Both the same
Neither

**If you have any further comments regarding the two visualisations shown above please add them here:**
[Empty text box for response]

**Supplementary Methods 2: Mixed Partial Proportional Odds Models (MPPOMs)**

Proportional odds models are typically used to estimate the cumulative probability of being in a given level of the Likert scale or lower[1]. They assume the relationship between all pairs of levels in the Likert scale are the same, i.e. the coefficients that describe the relationship between ‘disagree’ and all higher levels in the Likert scale are the same as those that describe the relationship between ‘somewhat disagree’ and all higher levels in the Likert scale[2].

If the assumption of proportional odds is met, only one set of coefficients must be estimated for each of the predictor variables[3]. However, if the assumption of proportional odds is not met, multiple sets of coefficients must be estimated to describe the relationship between each pair of levels. We used the nominal_test() function in the ordinal R package[4] to perform a Likelihood Ratio Test (LRT) of the proportional odds assumption for both the confidence and ease Likert scale data individually. As the nominal_test() function can only be used on a model that does not include random effects, we fit (non-mixed) proportional odds models to the confidence and ease data using the clm() function in the ordinal R package[4] before applying the nominal_test() function.

The LRTs indicated that background, level of education, and/or time of expertise did not meet the assumption of proportional odds for at least one of the measures of confidence and ease. We therefore fit MPPOMs to the confidence and ease Likert scale data, treating the predictor variables that did not meet the assumption of proportional odds as nominal effects.

**References**


1. Schmidt, J. Ordinal response mixed models: A case study. (Montana State University, Montana, USA, 2012)

2. Momeni, A. Pincus, M. & Libien, J. Introduction to statistical methods in pathology. (Springer, 2018).

3. Liu, X. Applied ordinal logistic regression using Stata: From single-level to multi-level modelling. (Sage Publications, 2015).

4. Christensen, R.H.B. ordinal - Regression models for ordinal data. R package version 2018.4-19. https://CRAN.R-project.org/package=ordinal Deposited 19 Apr 2018.

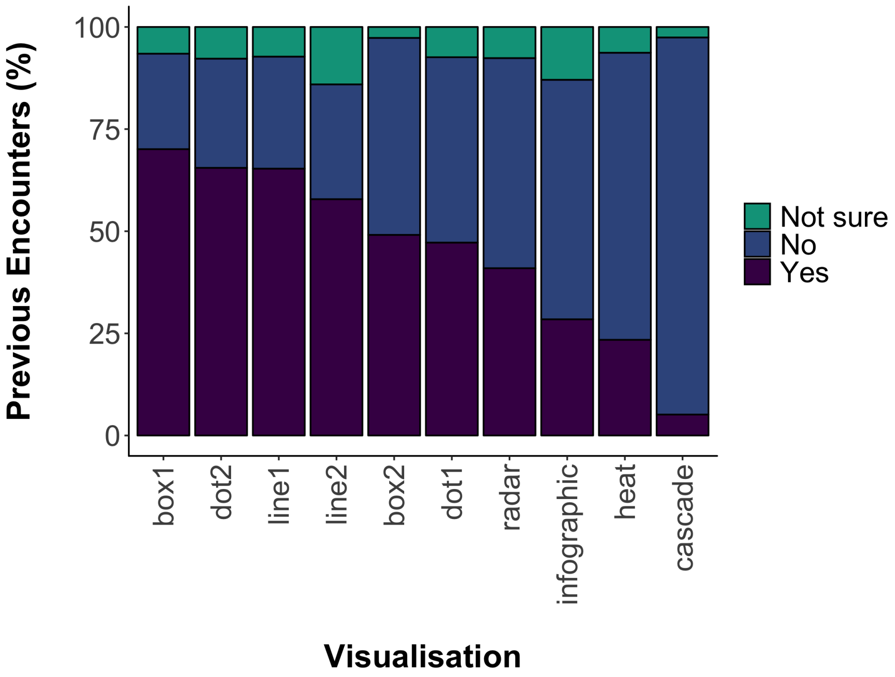


**Supplementary Figure 1** - The proportion of participants (%) that had previously encountered each visualisation type prior to completing the survey.

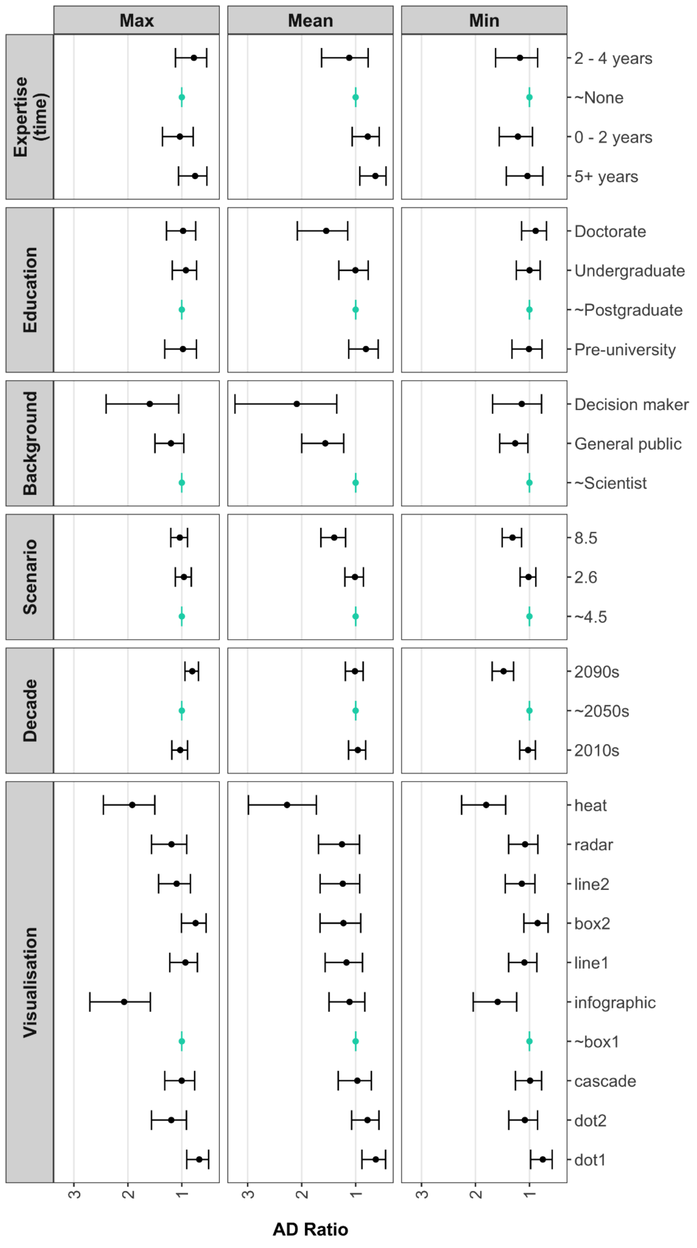


**Supplementary Figure 2** - The predictions (95% confidence interval) of the GLMMs that were used to analyse the absolute difference (x10) between the participants' estimates of the minimum (bottom), mean (middle), and maximum (top) projected temperature change and the true values given by the climate models. The reference levels associated with the 'typical' response are highlighted in teal and marked with a tilde.

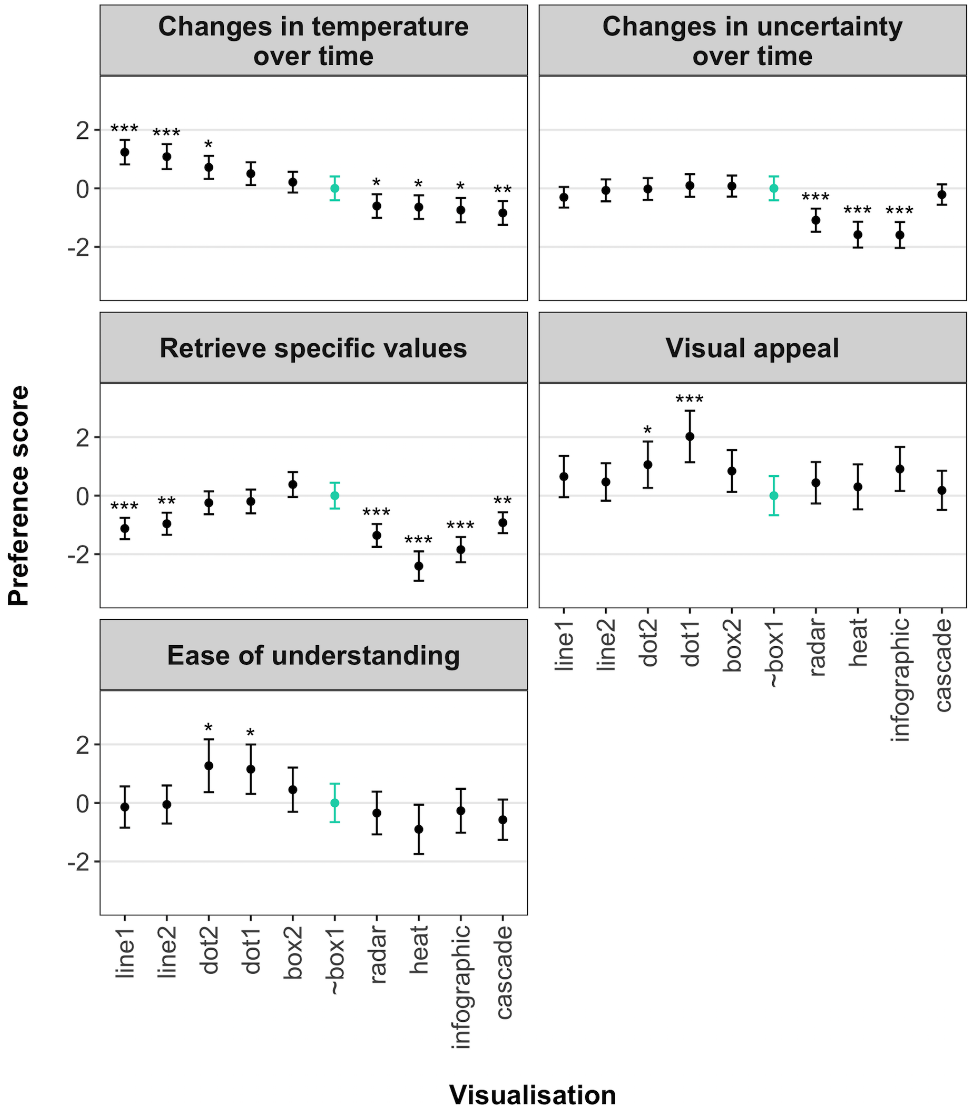


**Supplementary Figure 3** - The preference scores (95% 'comparison' intervals) of each visualisation across the five different preference categories. The preference scores are presented relative to the box1 plot (highlighted in teal and marked with a tilde), which was defined as the reference visualisation and is therefore given a preference score of zero. *p<0.05, **p<0.01, ***p<0.001.
